# Supplementary material for: Epitaxial growth of highly symmetrical branched noble metal-semiconductor heterostructures with efficient plasmon-induced hot-electron transfer
Source: Nat Commun. 2023 May 3;14:2538. doi: 10.1038/s41467-023-38237-7 (PMC10156852; doi:10.1038/s41467-023-38237-7)
Supplement: Supplementary file 1 — Supplementary Information [file 41467_2023_38237_MOESM1_ESM.pdf]

Supplementary Information

Epitaxial Growth of Highly Symmetrical Branched Noble Metal-Semiconductor Heterostructures with Efficient Plasmon-Induced Hot-Electron Transfer

Li Zhai[1,2,3,#], Sara T. Gebre[4,#], Bo Chen[2,#], Dan Xu[1,#], Junze Chen[5], Zijian Li[2], Yawei Liu[4], Hua Yang[2], Chongyi Ling[2], Yiyao Ge[2], Wei Zhai[2], Changsheng Chen[6], Lu Ma[7], Qinghua Zhang[8], Xuefei Li[1], Yujie Yan[9], Xinyu Huang[10], Lujiang Li[2], Zhiqiang Guan[2], Chen-Lei Tao[1], Zhiqi Huang[2], Hongyi Wang[2], Jinze Liang[2], Ye Zhu[6], Chun-Sing Lee[2], Peng Wang[9,11], Chunfeng Zhang[10], Lin Gu[12], Yonghua Du[7], Tianquan Lian[4,*], Hua Zhang[2,3,13,*], Xue-Jun Wu[1,*]

[1]State Key Laboratory of Coordination Chemistry, School of Chemistry and Chemical Engineering, Nanjing University, Nanjing 210023, China

[2]Department of Chemistry, City University of Hong Kong, Hong Kong, China

[3]Hong Kong Branch of National Precious Metals Material Engineering Research Center (NPMM), City University of Hong Kong, Hong Kong, China

[4]Department of Chemistry, Emory University, Atlanta, Georgia 30322, United States

[5]College of Materials Science and Engineering, Sichuan University, Chengdu, Sichuan 610065, China

[6]Department of Applied Physics, The Hong Kong Polytechnic University, Hung Hom, Hong Kong

[7]National Synchrotron Light Source II, Brookhaven National Laboratory, Upton, New York 11973, United States

[8]Beijing National Laboratory for Condensed Matter Physics, Institute of Physics, Chinese Academy of Sciences, Beijing 100190, China

[9]National Laboratory of Solid State Microstructures, Jiangsu Key Laboratory of Artificial Functional Materials, College of Engineering and Applied Sciences and Collaborative Innovation Center of Advanced Microstructures, Nanjing University, Nanjing 210093, China

[10]National Laboratory of Solid State Microstructures, School of Physics, and Collaborative Innovation Center of Advanced Microstructures, Nanjing University, Nanjing 210093, China

[11]Department of Physics, University of Warwick, Coventry CV4 7AL, United Kingdom

[12]Beijing National Center for Electron Microscopy and Laboratory of Advanced Materials, Department of Materials Science and Engineering, Tsinghua University, Beijing 100084, China

[13]Shenzhen Research Institute, City University of Hong Kong, Shenzhen 518057, China

[#]These authors contributed equally to this work: Li Zhai, Sara T. Gebre, Bo Chen, Dan Xu.

[*]Corresponding authors. E-mail: tlian@emory.edu; hua.zhang@cityu.edu.hk; xjwu@nju.edu.cn

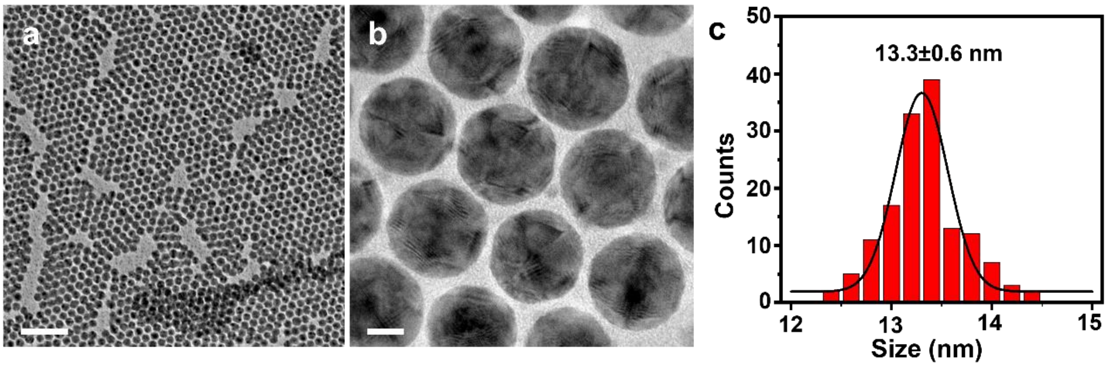

Supplementary Fig. 1. (**a**) Low-magnification TEM and (**b**) HRTEM images of Ag icosahedral nanocrystals. (**c**) Size distribution histogram of Ag icosahedral nanocrystals measured by TEM. (Scale bars: 100 nm in a; 5 nm in b).

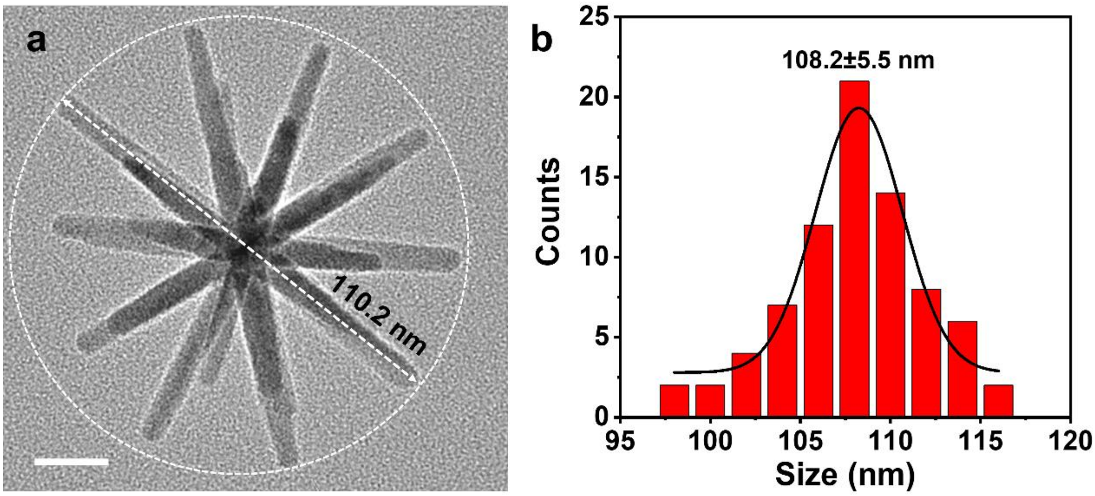

Supplementary Fig. 2. (**a**) HRTEM image of an individual Ag-CdS icosapod with its size indicated. (**b**) Size distribution histogram of Ag-CdS icosapods measured by TEM. (Scale bar: 20 nm in a).

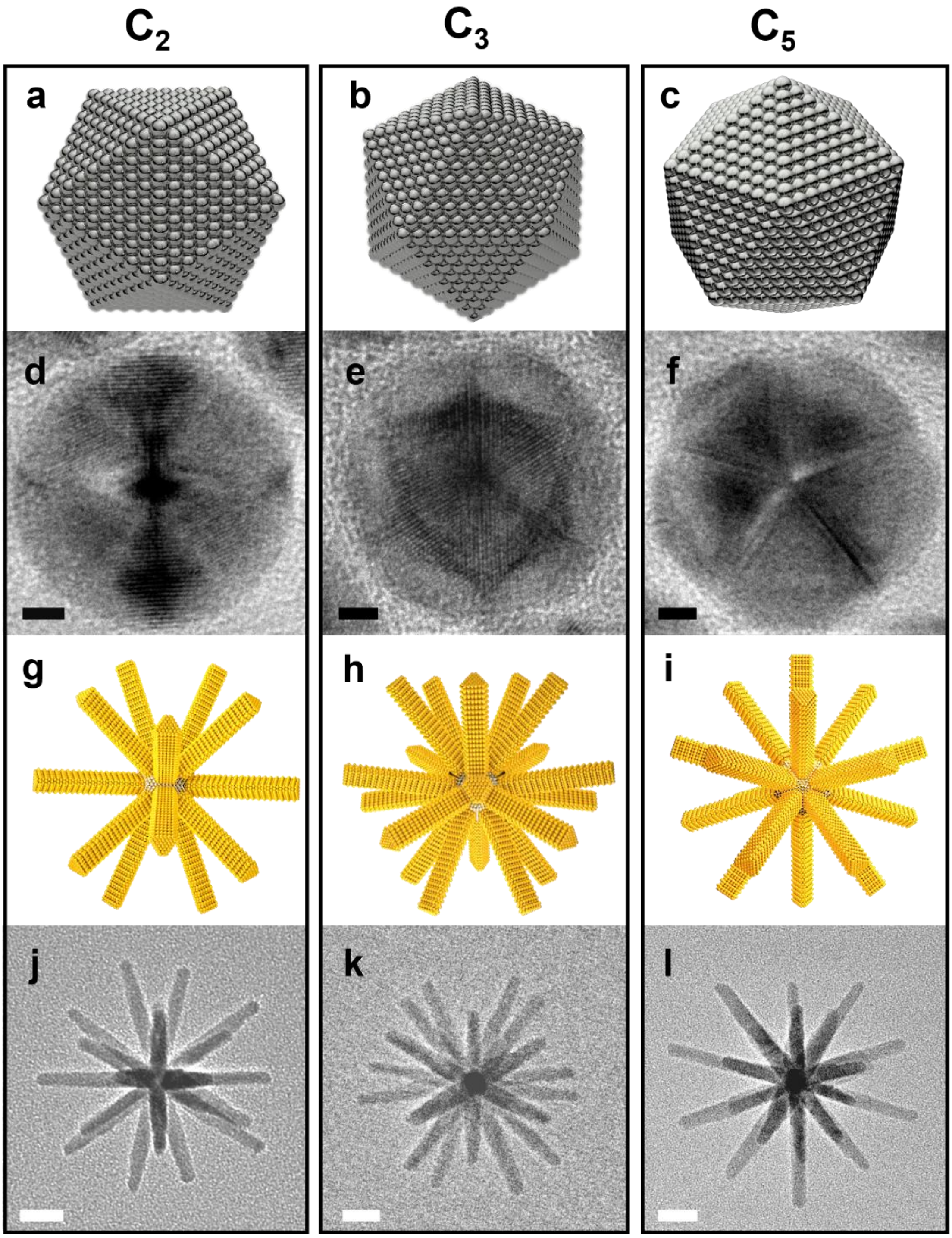

Supplementary Fig. 3. (**a** to **c**) Schematic illustrations and (**d** to **f**) HRTEM images of five-fold multi-twinned Ag icosahedral nanocrystals with C_2 (**a**,**d**), C_3 (**b**,**e**), and C_5 (**c**,**f**) symmetrical Moiré patterns. (**g** to **i**) Schematic illustrations and (**j** to **l**) HRTEM images of representative Ag-CdS icosapods with C_2 (**g**,**j**), C_3 (**h**,**k**), and C_5 (**i**,**l**) symmetrical patterns. (Scale bars: 2 nm in d to f; 20 nm in j to l).

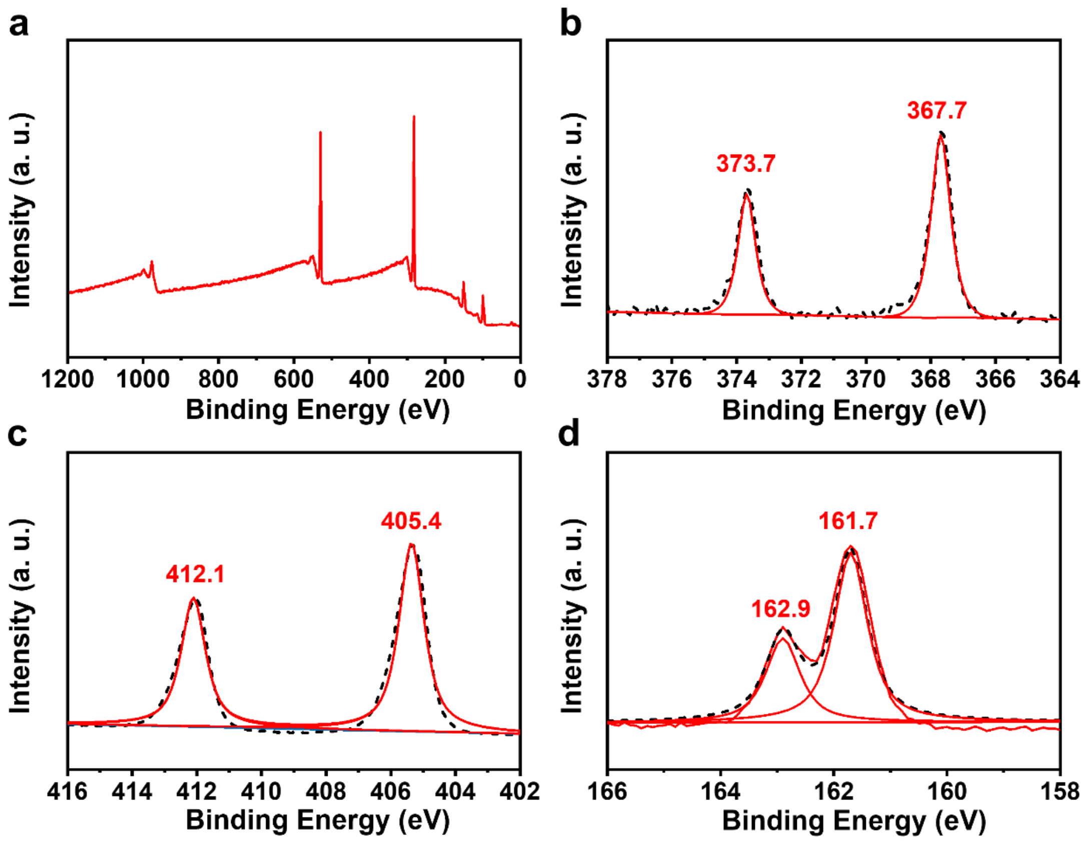

Supplementary Fig. 4. (**a**) XPS spectrum of the synthesized Ag-CdS icosapods. (**b**) High-resolution XPS spectrum of *Ag 3d*. (**c**) High-resolution XPS spectrum of *Cd 3d*. The peaks located at 412.1 and 405.4 eV can be ascribed to $Cd\ 3d_{3/2}$ and $Cd\ 3d_{5/2}$ of CdS, respectively. (**d**) High-resolution XPS spectrum of *S 2p*. The peaks located at 162.9 and 161.7 eV can be ascribed to $S\ 2p_{1/2}$ and $S\ 2p_{3/2}$ of CdS, respectively[1,2].

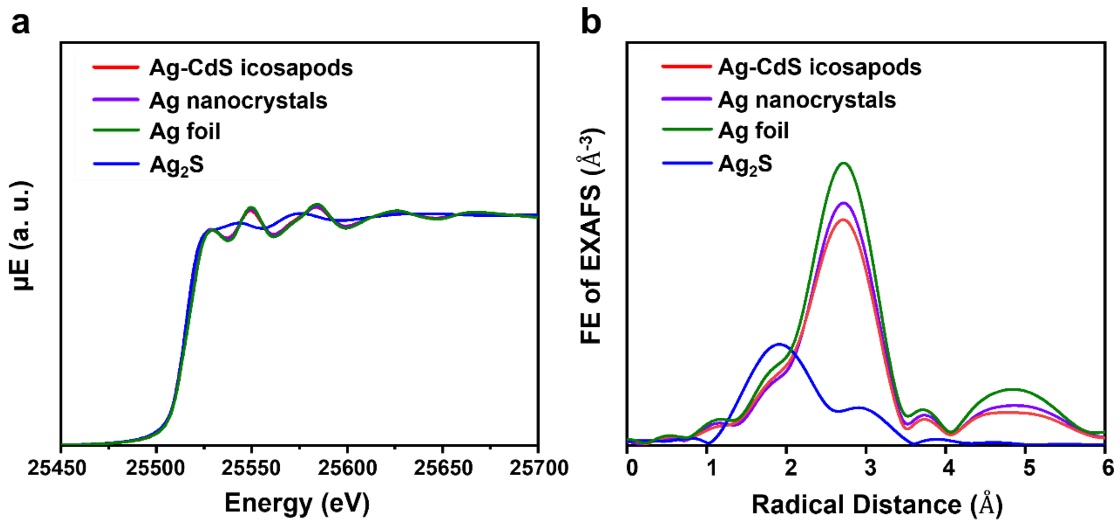

Supplementary Fig. 5. (**a**) Normalized Ag K-edge XANES spectra and (**b**) Fourier-transformed EXAFS spectra of the Ag-CdS icosapods, Ag icosahedral nanocrystals, Ag foil and Ag_2S nanocrystals.

Supplementary Note 1: As shown in Supplementary Fig. 5a, in the Ag K-edge, the white line intensity and XANES energy of Ag-CdS icosapods match well with those of Ag nanocrystals and Ag foil. And in the Fourier transforms of EXAFS spectra (Supplementary Fig. 5b), Ag-CdS icosapods, Ag nanocrystals and Ag foil all show a dominant peak at approximately 2.85 Å in R space, corresponding to the Ag-Ag scattering path[3]. The XANES spectra and Fourier transformed EXAFS spectra of Ag-CdS icosapods are different from those of Ag_2S, demonstrating that the Ag in Ag-CdS icosapods was not sulfurized. The Ag_2S nanocrystals were synthesized according to the previous report[2].

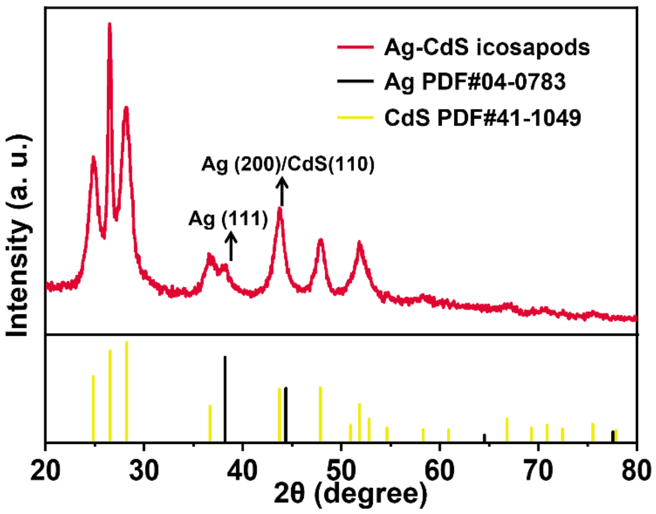

Supplementary Fig. 6. XRD pattern of the synthesized Ag-CdS icosapods. The standard diffraction peaks of wurtzite CdS (yellow lines, PDF#41-1049) and *fcc* Ag (black lines, PDF#04-0783) are used as references.

Supplementary Note 2: As shown in Supplementary Fig. 6, the peaks located at 24.9°, 26.5°, 28.2°, 36.6°, 43.7°, 47.8° and 51.9 °are well matched with the (100), (002), (101), (102), (110), (103) and (112) planes of wurtzite CdS, respectively. In addition, the peak located at 38.2° corresponds to the (111) plane of Ag, and the peak of Ag (200) plane located at 44.2° is overlapped with the (110) plane of CdS.

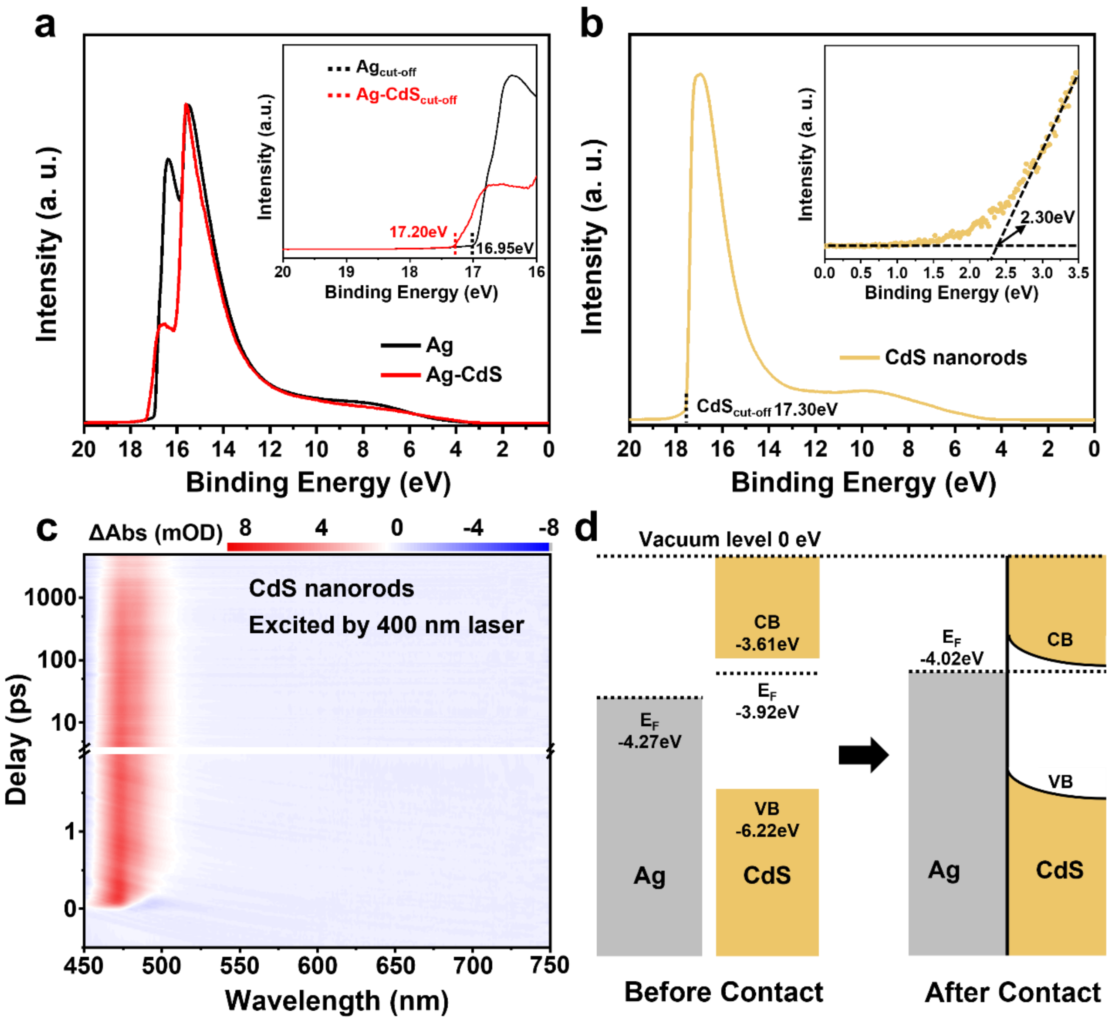

Supplementary Fig. 7. (**a**) UPS spectra of Ag nanocrystals and Ag-CdS icosapods. Inset: the zoom-in UPS spectra of Ag nanocrystals and Ag-CdS icosapods. (**b**) UPS spectra of CdS nanorods. Inset: the zoom-in UPS spectra of CdS nanorods. (**c**) Two-dimensional pseudo-color plots of pump-visible probe TA spectra of the colloid solution of CdS in toluene pumped by 400 nm laser. (**d**) The schematic illustration of the interfacial Schottky barrier in the Ag-CdS icosapods.

Supplementary Note 3: As shown in Supplementary Fig. 7a, the cut-off energy of Ag nanocrystals and Ag-CdS icosapods is determined to be 16.95 eV and 17.20 eV, respectively. The corresponding work functions (Φ) can be estimated according to the following equation: $\Phi = h\nu - |E_{cut\text{-}off} - E_f|$, where $h\nu$ was the fixed incident photon energy of 21.22 eV (He I lamp) and E_f was calibrated to 0 eV using a standard Au sample. Φ can be estimated to be 4.27 eV for Ag nanocrystals and 4.02 eV for Ag-CdS icosapods. Here, the Fermi level of Ag nanocrystals and Ag-CdS icosapods should be -4.27 eV and

-4.02 eV relative to the vacuum level, respectively. The cut-off energy of CdS nanorods is determined to be 17.30 eV. Therefore, the Fermi level of CdS should be -3.92 eV relative to the vacuum level (Supplementary Fig7b). The distance between Fermi level and valence band (VB) of CdS is measured to be 2.30 eV by the linear fitting of the UPS spectrum in the long tail (inset in Supplementary Fig. 7b), demonstrating the valence band (VB) of CdS nanorods is -6.22 eV. Meanwhile, the bandgap of CdS nanorods can be obtained from bleach signal of CdS with center located at ~475 nm (2.61 eV) in the ultrafast pump-probe TA spectrum (Supplementary Fig. 7c). Therefore, the conduction band (CB) of CdS should be -3.61 eV. Based on the results, the interfacial electronic structure can be schematically shown in Supplementary Fig. 7d. The shift (~0.25 eV) of the Fermi level in Ag-CdS icosapods compared with that of Ag nanocrystals demonstrates a Schottky barrier was formed at the Ag-CdS interface[4].

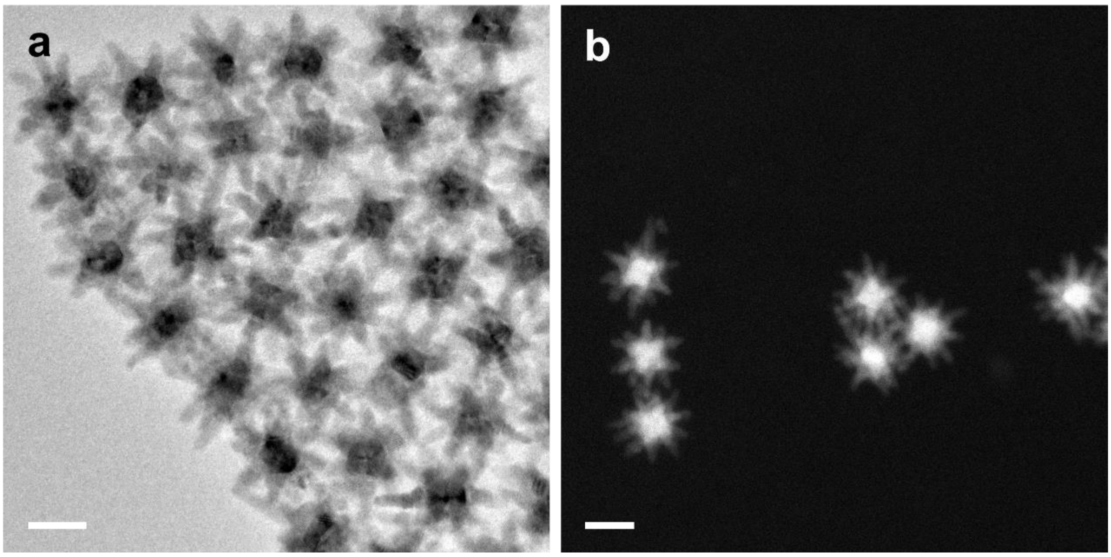

Supplementary Fig. 8. (**a**) TEM and (**b**) HAADF-STEM images of the synthesized Ag-CdSe icosapods, showing uniform size distribution with the CdSe length of ~15 nm and Ag core of ~14 nm. (Scale bars: 20 nm).

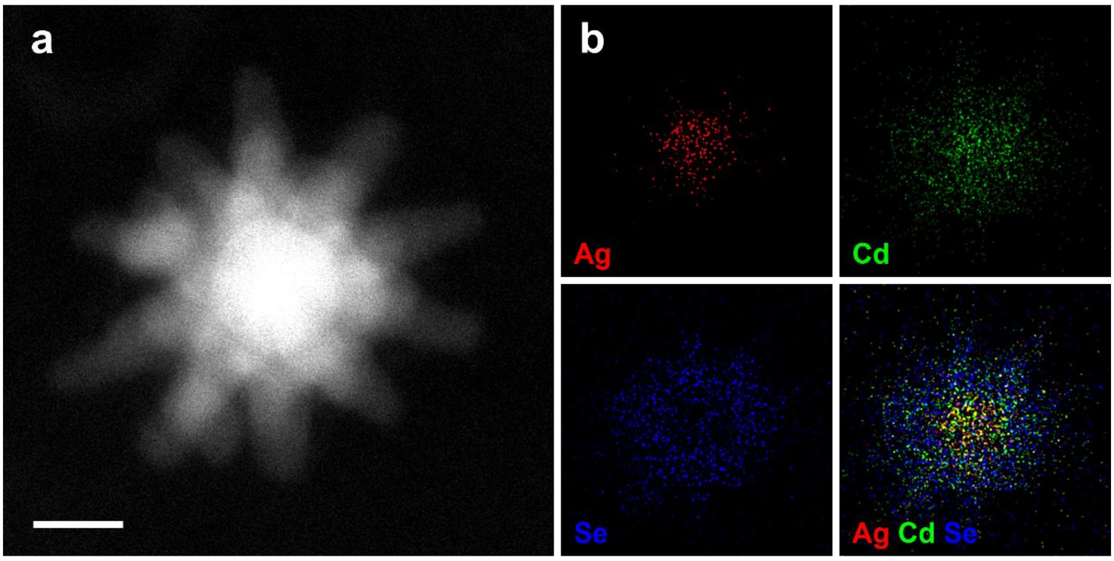

Supplementary Fig. 9. (**a**) HAADF-STEM and (**b**) the corresponding EDS mapping images of a representative Ag-CdSe icosapod, showing the Cd and Se elements distributed homogeneously on the nanorods and the Ag element located in the core. (Scale bars: 20 nm).

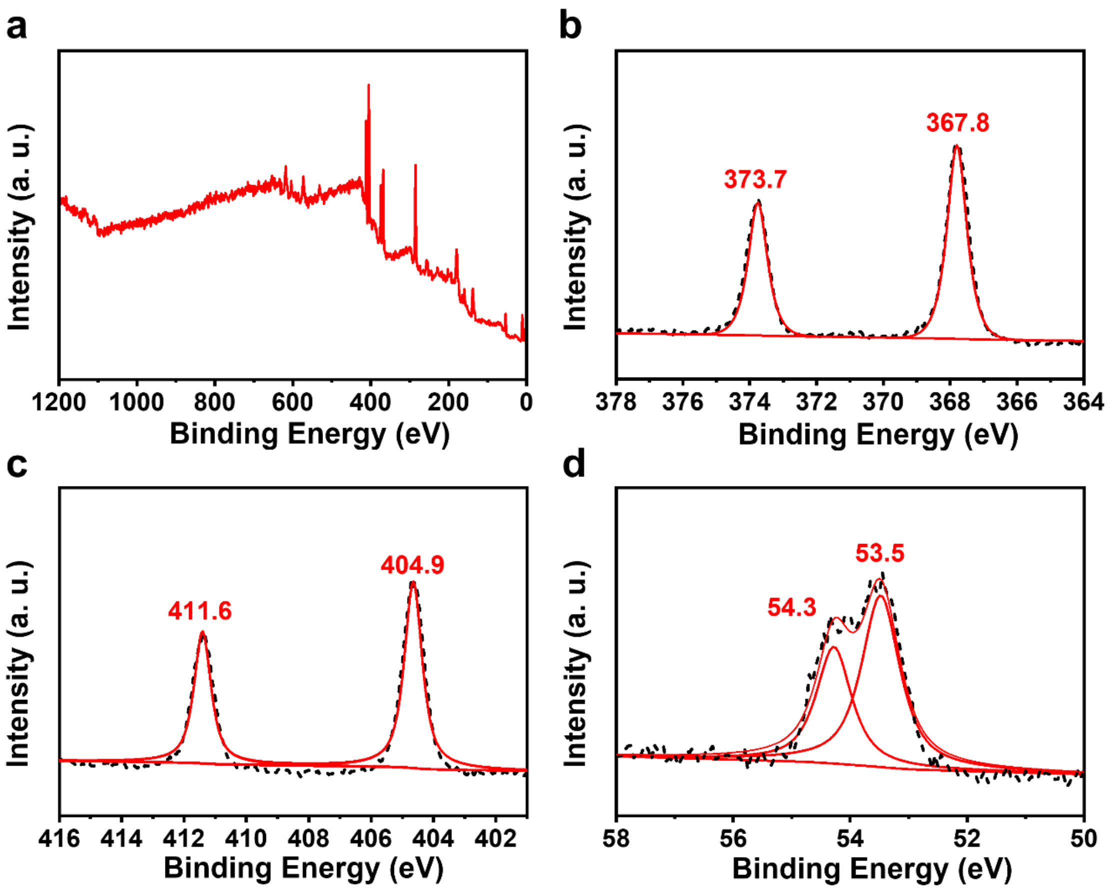

Supplementary Fig. 10. (**a**) XPS spectrum of the synthesized Ag-CdSe icosapods. (**b**) High-resolution XPS spectra of *Ag 3d*. The peaks located at 373.7 and 367.8 eV can be ascribed to $Ag\ 3d_{3/2}$ and $Ag\ 3d_{5/2}$ of zerovalent Ag, respectively. (**c**) High-resolution XPS spectrum of *Cd 3d*. The peaks located at 411.6 and 404.9 eV can be ascribed to $Cd\ 3d_{3/2}$ and $Cd\ 3d_{5/2}$ of CdSe, respectively. (**d**) High-resolution XPS spectrum of *Se 2p*. The peaks located at 54.3 and 53.5 eV can be ascribed to $Se\ 2d_{3/2}$ and $Se\ 2d_{5/2}$ of CdSe, respectively[5].

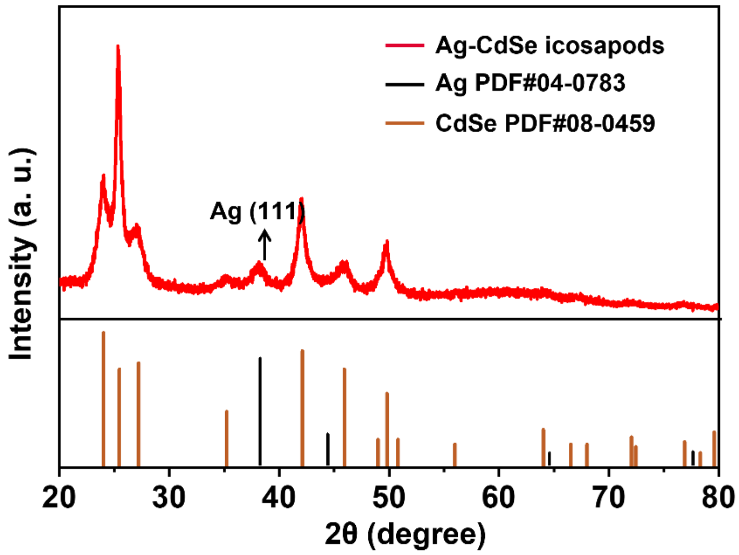

Supplementary Fig. 11. XRD pattern of the synthesized Ag-CdSe icosapods. The standard diffraction peaks of CdSe (brown lines, PDF#08-0459) and *fcc*-Ag (black lines, PDF#04-0783) are used as references.

Supplementary Note 4: As shown in Supplementary Fig. 11, the XRD pattern of the synthesized Ag-CdSe icosapods can be well indexed to the wurtzite CdSe and *fcc*-Ag. The peak located at 38.2° corresponds to the (111) plane of Ag. The peaks located at 23.9°, 25.4°, 27.1°, 35.1°, 42.0°, 45.8° and 49.7° are well matched with the (100), (002), (101), (102), (110), (103) and (112) planes of wurtzite CdSe, respectively.

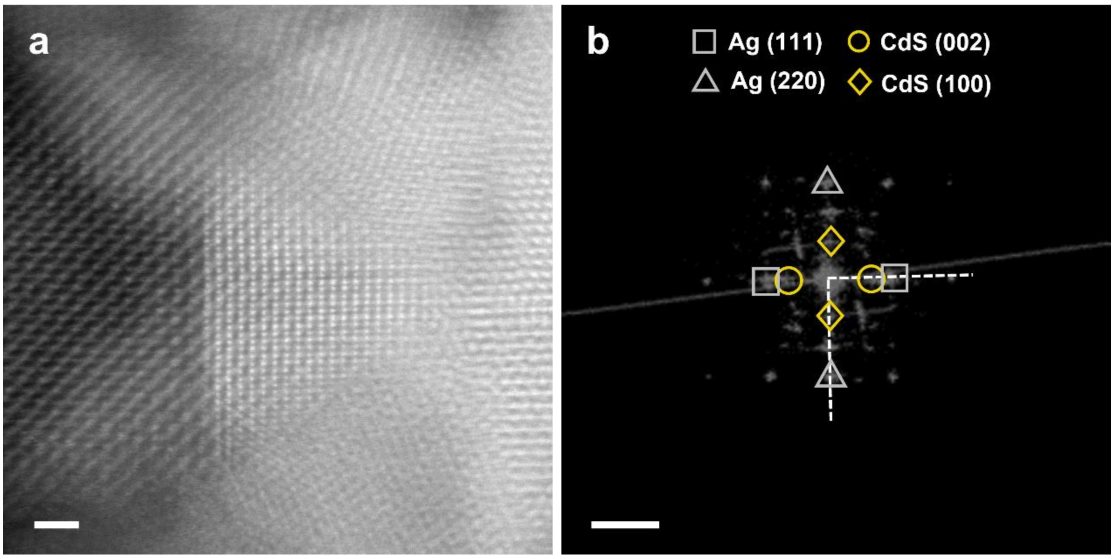

Supplementary Fig. 12. (**a**) Cs-HAADF-STEM image and (**b**) the corresponding FFT pattern of the interface in Ag-CdS icosapods. (Scale bars: 1 nm in a; 5 1/nm in b).

Supplementary Note 5: Due to the sophisticated symmetrical branched architecture of Ag-CdS icosapods with a large size of 108.2±5.5 nm (Fig. 1b-g and Supplementary Fig. 2), it is very difficult to clearly observe the detailed structure of the interfaces in Ag-CdS icosapods (Supplementary Fig. 12a). However, an epitaxial relationship can be found between Ag (111) and CdS (002) planes, as confirmed by the corresponding FFT pattern (Supplementary Fig. 11b).

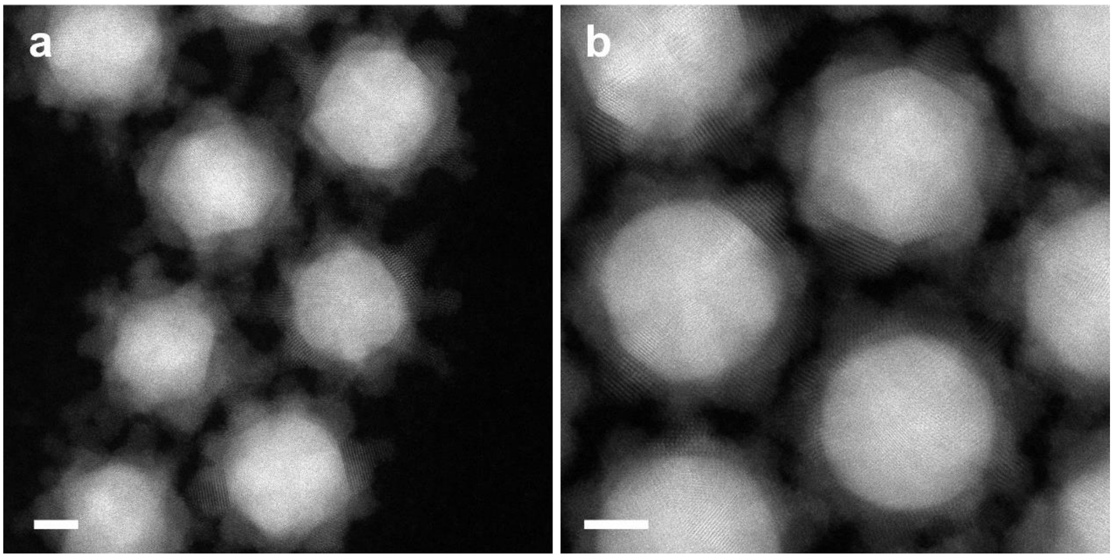

Supplementary Fig. 13. Cs-HAADF-STEM images of the (**a**) Ag-CdS and (**b**) Ag-CdSe heterostructures were obtained at a short reaction time of 1 min. The obtained Ag-CdS and Ag-CdSe heterostructures were used for the characterization of the epitaxial interfaces shown in Fig. 2. (Scale bars: 5 nm).

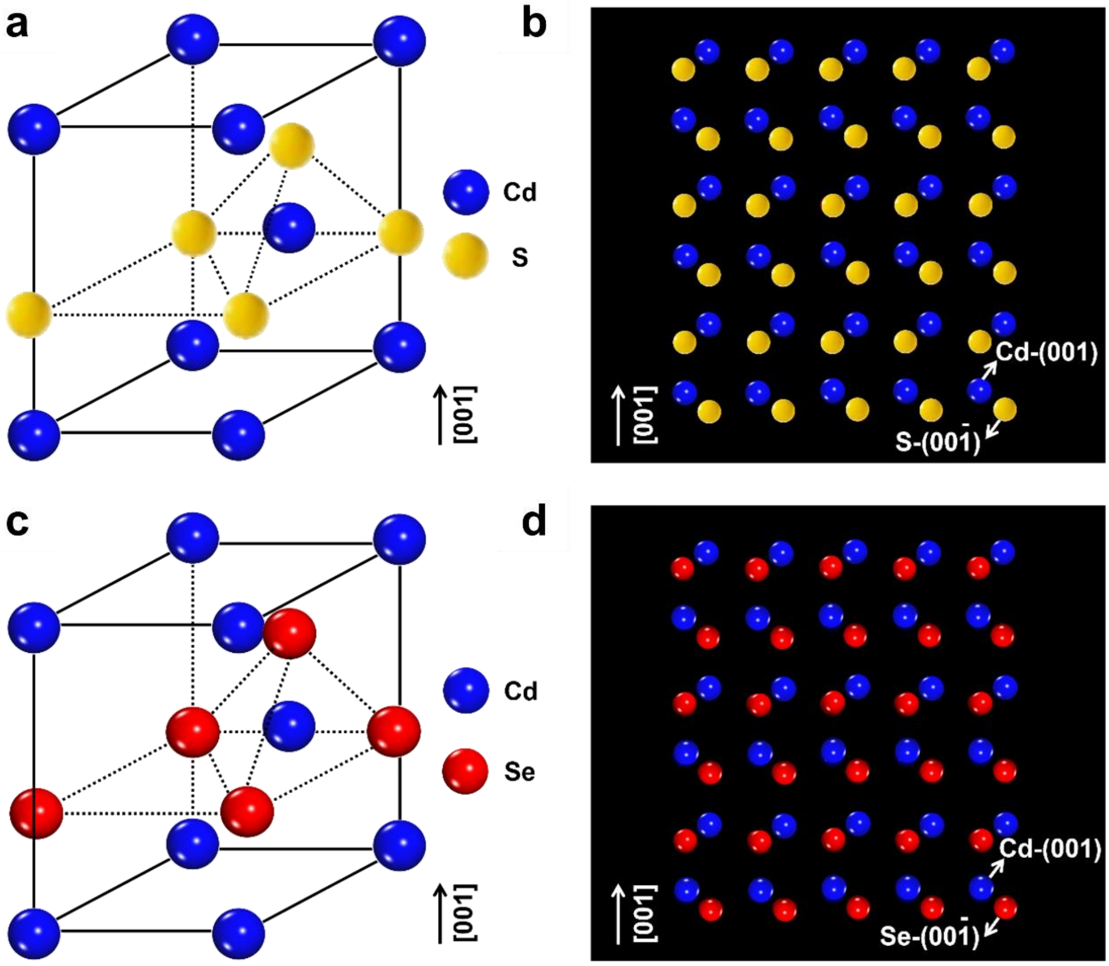

Supplementary Fig. 14. (**a**) Schematic crystal model of wurtzite CdS. (**b**) Schematic lattice model of wurtzite CdS along its [001] direction. (**c**) Schematic crystal model of wurtzite CdSe. (**d**) Schematic lattice model of wurtzite CdSe along its [001] direction.

Supplementary Note 6: The wurtzite CdS exhibits crystallographic polarity along its c-axis, which constitutes alternating Cd and S atom layers along its [001] direction (Supplementary Fig. 14a). Such polar crystal has two kinds of polarized surface, *i.e.*, Cd-terminated (001) planes and S-terminated $(00\bar{1})$ planes (Supplementary Fig. 14b). Similarly, the wurtzite CdSe exhibits crystallographic polarity along its c-axis, which constitutes alternating Cd and Se atom layers along its [001] direction (Supplementary Fig. 14c). Such polar crystal has two kinds of polarized surface, *i.e.*, Cd-terminated (001) planes and Se-terminated $(00\bar{1})$ planes (Supplementary Fig. 14d).

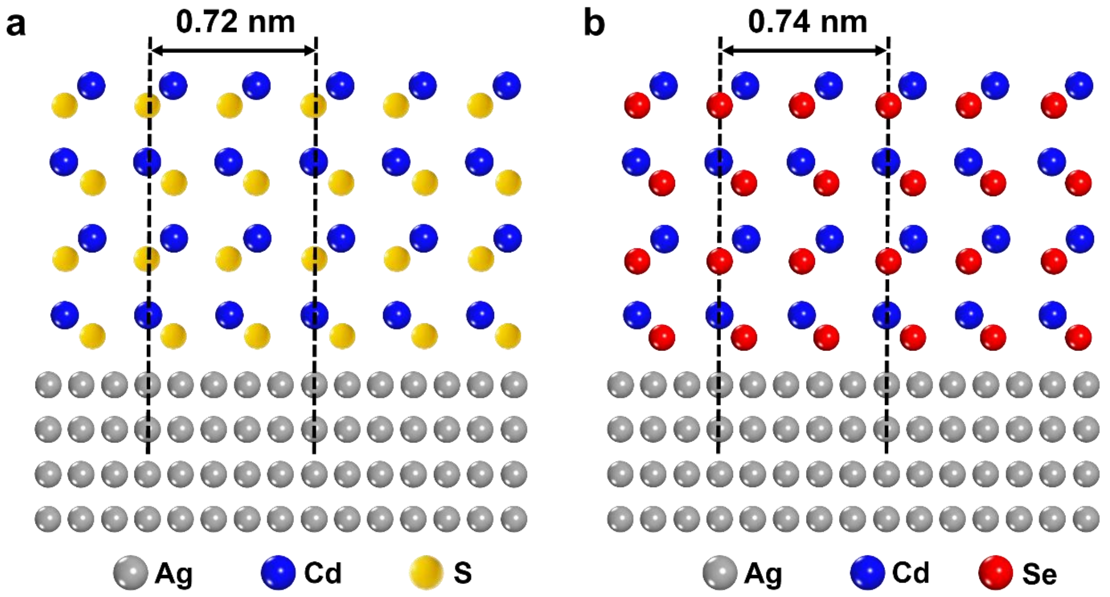

Supplementary Fig. 15. Schematic lattice structures of the epitaxial (**a**) Ag-CdS interface and (**b**) Ag-CdSe interface.

Supplementary Note 7: As schematically shown in Supplementary Fig. 15, the lattice distance of two (002) planes of CdS (0.72 nm) is similar to that of five (220) planes of Ag (0.72 nm), and the lattice distance of two (002) planes of CdSe (0.74 nm) is also similar to that of five (220) planes of Ag (0.72 nm).

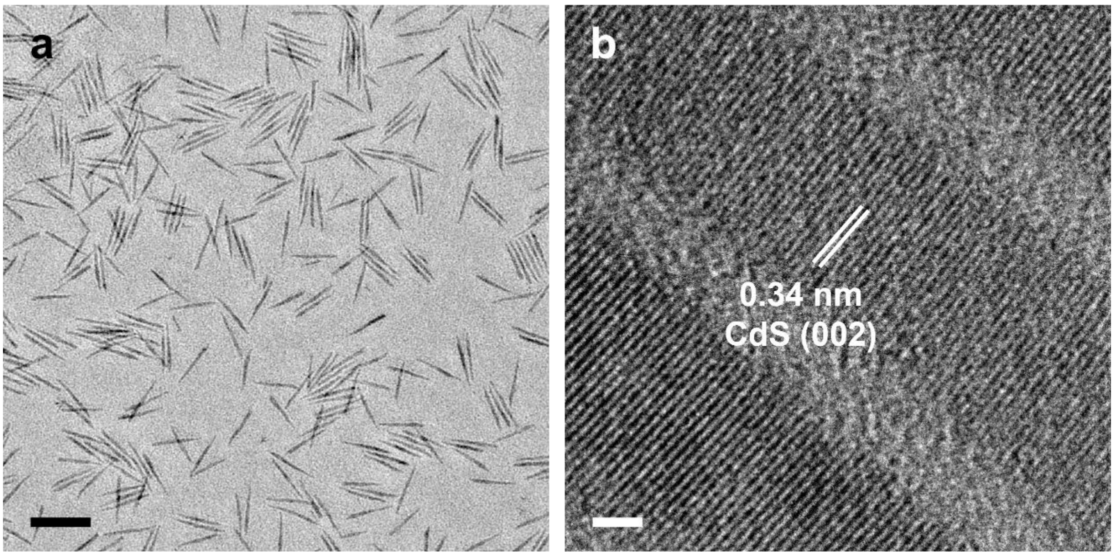

Supplementary Fig. 16. (**a**) TEM and (**b**) HRTEM images of CdS nanorods. (Scale bars: 50 nm in a; 2 nm in b).

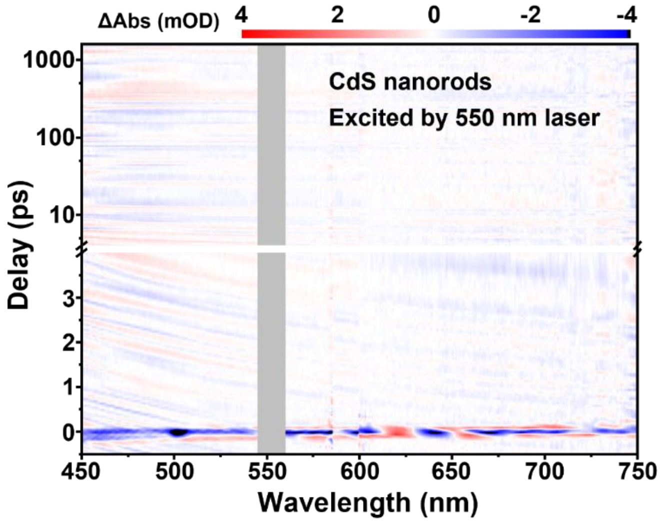

Supplementary Fig. 17. Two-dimensional pseudo-color plots of pump-visible probe TA spectra of the colloid solution of CdS in toluene pumped by 550 nm laser. Grey shading is used to avoid the disturbance of the 550 nm pump laser to the probed signal.

Supplementary Note 8: When the CdS nanorods were pumped by a 550 nm laser, no obvious bleaching signal was observed, confirming that the 550 nm laser could not excite CdS nanorods.

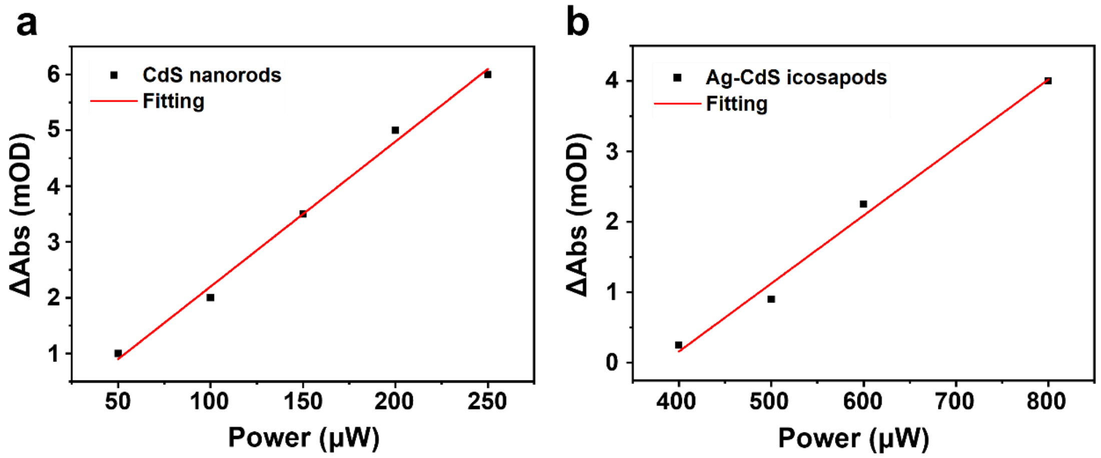

Supplementary Fig. 18. The power-dependence absorption plots of (**a**) CdS nanorods and (**b**) Ag-CdS icosapods. The slope values are listed in Tables S2.

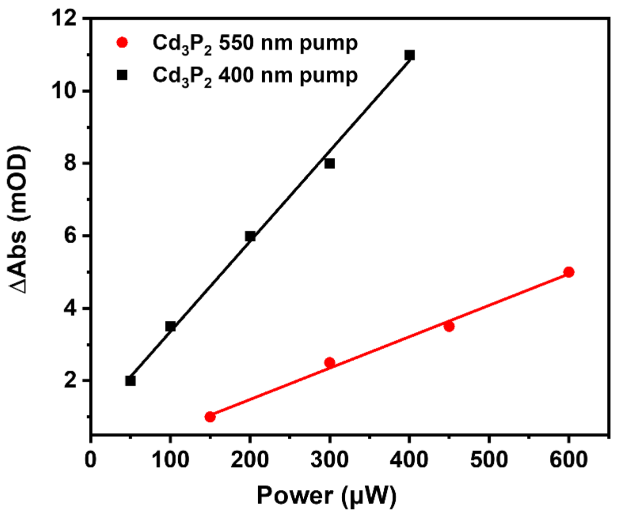

Supplementary Fig. 19. The power-dependence absorption plots of Cd_3P_2. The slope values are listed in Tables S2.

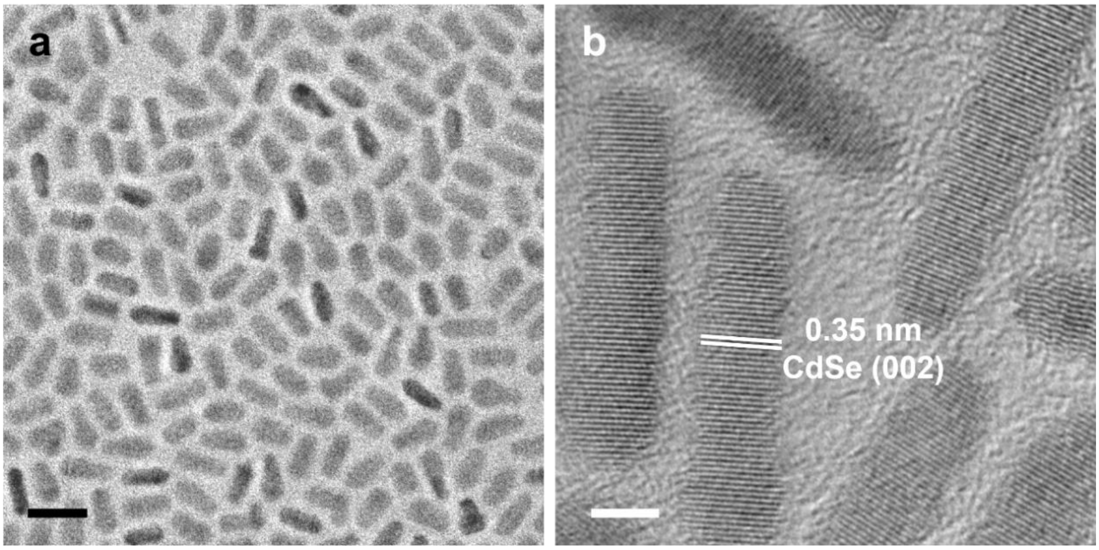

Supplementary Fig. 20. (**a**) TEM and (**b**) HRTEM images of CdSe nanorods. (Scale bars: 20 nm in a; 2 nm in b).

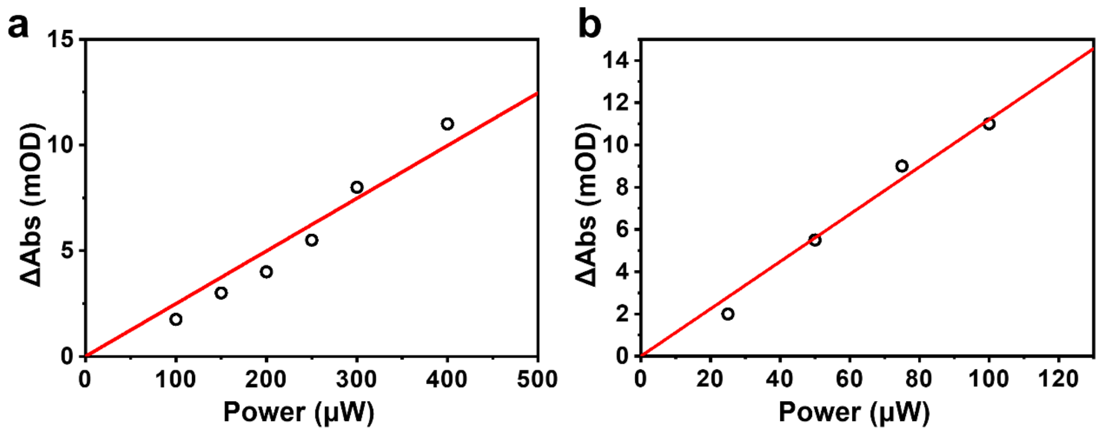

Supplementary Fig. 21. The power-dependence absorption plots of (**a**) CdSe nanorods and (**b**) Ag-CdSe icosapods. The slope values are listed in Tables S4.

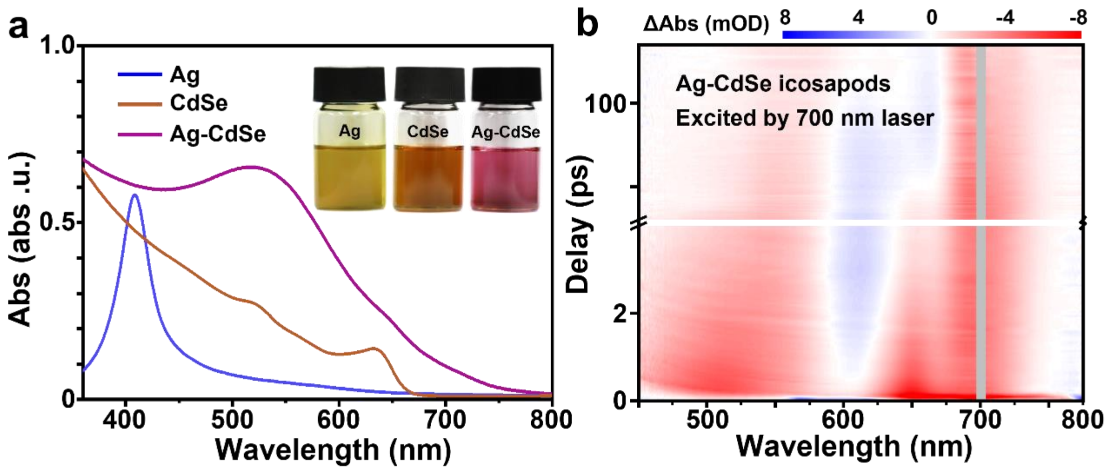

Supplementary Fig. 22. (**a**) UV-visible absorption spectra of the colloidal solutions of Ag icosahedral nanocrystals, CdSe nanorods and Ag-CdSe icosapods in toluene. Insets: photographs of the corresponding colloid solutions used for UV-visible absorption measurements. (**b**) Two-dimensional pseudo-color plots of pump-visible probe TA spectra of the colloid solutions of Ag-CdSe in toluene. Grey shading is used to avoid the disturbance of the 700 nm pump laser to the probed signal. Source data are provided as a Source Data file.

Supplementary Note 9: As shown in Supplementary Fig. 22a, the SPR peak of Ag-CdSe icosapods shifts from 408 nm (blue curve) to 550 nm and merges with the CdSe nanorod exciton bands (brown curve) to form a broad absorption feature extending to ~800 nm (fuchsia curve). Compared with the Ag nanocrystals and CdSe nanorods, the absorption ranging from 675 to 800 nm could also be attributed to the broadened and redshifted Ag SPR band. When the Ag-CdSe icosapods were excited by a 700 nm pump laser, a strong bleaching signal near 650 nm and a broad bleaching signal ranging from 450 to 600 nm are observed (Supplementary Fig. 22b), which can also be attributed to the plasmon-induced hot-electron transfer from the Ag core to CdSe nanorods.

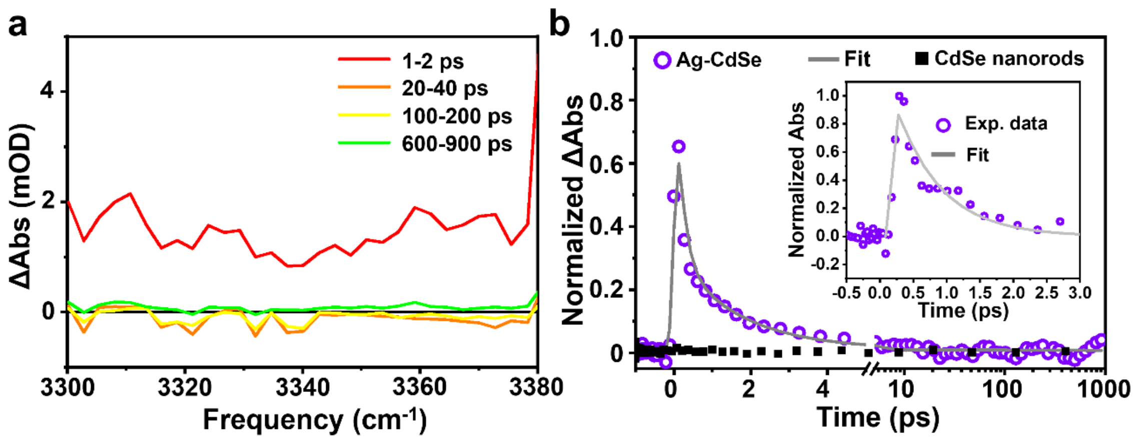

Supplementary Fig. 23. (**a**) Averaged mid-IR TA spectra of Ag-CdSe icosapods from 1 to 2 ps (red curve), 20 to 40 ps (orange curve), 100 to 200 ps (yellow curve), and 600 to 900 ps (green curve) after 700 nm laser excitation. (**b**) Time-resolved mid-IR absorption spectrum of Ag-CdSe icosapods. Grey curves represent the multiexponential fits as described in Supplementary Table 3. A negligible intraband absorption signal (black squares) is observed in a control experiment conducted on CdSe nanorods. Inset: zoom-in time-resolved mid-IR absorption spectrum of Ag-CdSe icosapods. Source data are provided as a Source Data file.

Supplementary Note 10: In the time-resolved absorption spectrum of Ag-CdSe icosapods (Supplementary Fig. 23b), the sharply increased TA signal corresponds to the plasmon-induced hot-electron transfer from Ag into CdSe, and the TA signal decay corresponds to the charge-recombination process. The fitting result (Supplementary Table 3) demonstrates an ultrafast plasmon-induced hot-electron transfer time from the Ag to CdSe (about 7.5 fs).

Supplementary Table 1. Summary of lattice mismatches of Ag and CdS in Ag-CdS icosapods, and Ag and CdSe in Ag-CdSe icosapods.

Lattice parameter of noble metal (LM)	Ag (111) 0.236 nm	Ag (111) 0.236 nm	Ag (220) 0.144 nm	Ag (220) 0.144 nm
Lattice parameter of semiconductor (LS)	CdS (002) 0.336 nm	CdSe (002) 0.351 nm	CdS (100) 0.359 nm	CdSe (100) 0.372 nm
Lattice mismatch	42.4%	48.7%	149.3%	158.3%

The mismatches are calculated by the conventional definition6 as Eq. S1:

$$\text{Lattice mismatch} = \frac{\text{LS}-\text{LM}}{\text{LM}} * 100\% \qquad (1)$$

Supplementary Table 2. Parameters used for calculating the QY of Ag-CdS icosapods according to Eq. 4.

	CdS excited by 400 nm laser	Cd_3P_2 excited by 400 nm laser	Ag-CdS excited by 550 nm laser	Cd_3P_2 excited by 550nm laser
ΔS (mOD μW^{-1})	0.026	0.025	0.0097	0.030
OD	0.19	0.84	0.62	0.29
QY	100%	N/A	18.1%	N/A

Abbreviation: N/A, Not applicable.

Supplementary Table 3. Fitting results of the intraband absorption kinetics of Ag-CdS and Ag-CdSe icosapods according to Eq. 6.

	T_f	i=1	i=2	i=3
Ag-CdS	18.0 ± 0.3 fs	1.9 ± 0.16 ps	52.4 ± 16.6 ps	1347.5 ± 385 ps
Ag-CdSe	7.5 ± 7.2 fs	0.2 ± 0.1 ps	1.7 ± 0.3 ps	

Supplementary Table 4. Parameters used for calculating the QY of Ag-CdSe icosapods according to Eq. 5.

	CdSe excited by 400 nm laser	Cd_3P_2 excited by 400 nm laser	Ag-CdSe excited by 700 nm laser	Cd_3P_2 excited by 700 nm laser
ΔS (mOD μW^{-1})	0.025	0.032	0.112	0.557
OD	0.43	0.68	0.48	0.34
QY	100%	N/A	17.6%	N/A

Abbreviation: N/A, Not applicable.

Supplementary references

1. Stoev, M. & Katerski, A. XPS and XRD study of photoconductive CdS films obtained by a chemical bath deposition process. *J. Mater. Chem.* **6**, 377-380 (1996).

2. Chen, J. Z. *et al.* Ag@MoS_2 core-shell heterostructure as SERS platform to reveal the hydrogen evolution active sites of single-layer MoS_2. *J. Am. Chem. Soc.* **142**, 7161-7167 (2020).

3. Huang, J. *et al.* Energy-dispersive X-ray absorption spectroscopy with an inverse compton source. *Sci. Rep.* **10**, 8772 (2020).

4. Li Wang *et al.* $CuSbS_2$ solar cells using CdS, In_2S_3 and the In/Cd-based hybrid buffers. *J. Electron. Mater.* **50**, 3283-3287 (2021).

5. Katari, J. E. B., Colvin, V. L. & Alivisatos, A. P. X-ray photoelectron-spectroscopy of CdSe nanocrystals with applications to studies of the nanocrystal surface. *J. Phys. Chem.* **98**, 4109-4117 (1994).

6. Zhang, J. T., Tang, Y., Lee, K. & Min, O. Y. Nonepitaxial growth of hybrid core-shell nanostructures with large lattice mismatches. *Science* **327**, 1634-1638 (2010).
