# Supplementary material for: Epitaxial growth of highly symmetrical branched noble metal-semiconductor heterostructures with efficient plasmon-induced hot-electron transfer
Source: Nat Commun. 2023 May 3;14:2538. doi: 10.1038/s41467-023-38237-7 (PMC10156852; doi:10.1038/s41467-023-38237-7)
Supplement: Supplementary file 3 — Description of Additional Supplementary Files [file 41467_2023_38237_MOESM3_ESM.pdf]

**Description of Additional Supplementary Files**

File Name: Supplementary  Data 1
Description: Interface Structure: The 3D model of the epitaxial Ag-CdS interface.

File Name: Supplementary Movie 1
Description: Interface Structure: The 3D model of the epitaxial Ag-CdS interface.
